# The Status of Obstetrics

**Published:** 1901-12

**Authors:** 


### The Status of Obstetrics.

Dr. David S. Funk, in the *Philadelphia Medical Journal,* has a lengthy and interesting paper on several of the most serious obstetrical complications and operative procedures.

Placenta prævia, he says, will, for many years to come, remain one of the most alarming obstetrical complications, because there can be no prophylaxis, and because of the appalling rapidity with which it destroys life. The condition, he claims, is much less frequent than is commonly taught. In reviewing the treatment in vogue today he thinks no real advances have been made during the past three hundred years. The time for emptying the uterus is as soon as the diagnosis is made. The popular method of manual dilatation with version does not always lessen the danger to either mother or child.

Symphyseotomy is, in this country, being displaced by cæsarean section in those cases in which the disproportion between the child and pelvic outlet is of such character as to render high forceps operation impossible or dangerous. The mortality of cæsarean section is now lower than the mortality in symphyseotomy, and the convalescence much shorter.

With reference to septicæmia, he thinks the proper treatment is disinfection of the vagina, exploration of the uterine cavity, followed by intra-uterine douching with normal salt solution at 110° F., and packing with iodoform gauze. In some cases of true septicæmia the intravenous injection of normal salt solution is followed by good results.

Ectopic gestation is now recognized to be of comparatively common occurrence, and the general practitioners should be on the lookout for it. In all cases giving a previous history of sterility in which there is a sudden cessation of a previously regular menstruation, irregular uterine bleeding, sharp, collicky pains and the other symptoms and signs suggestive of extrauterine pregnancy, a bimanual examination should be insisted upon. R.

